# Combined Non-Invasive Cardiac Imaging and Genetic Testing of Elite Volleyball Players: A Ten-Year Experience

**DOI:** 10.26502/fccm.92920220

**Published:** 2021-10-08

**Authors:** Paul Grossfeld

**Affiliations:** Division of Cardiology, Department of Pediatrics, UCSD School of Medicine, La Jolla, California

**Keywords:** Marfan syndrome, Sinuses of Valsalva, Volleyball, Fibrillin-1

## Abstract

Sudden cardiac death in athletes is a devastating event. Although significant progress has been made in identifying the underlying pathophysiology and genetic basis for sudden cardiac death in young athletes, controversy exists regarding cost-effective screening measures to identify at-risk individuals. In this report we describe our ten-year experience performing cardiovascular assessments on 150 members of the United States Men’s and Women’s National Volleyball teams. Through a combination of history, physical, echocardiography and genetic testing, we have identified one previously undiagnosed athlete with Marfan syndrome, along with four others with a possible aortopathy. Taken together, this approach is a cost-effective strategy for the identification of at-risk tall athletes leading to potentially lifesaving interventions, and raises the issue of the feasibility of screening for all tall individuals.

## 1. Introduction

On January 24, 1986 31 year-old Flo Hyman, one of the top female volleyball players in the world, collapsed and died from an acute aortic dissection during a competitive volleyball match in Matsue, Shimane, Japan. She was subsequently found to have Marfan syndrome. Sudden cardiac death in young athletes is a relatively rare but devastating event for which there have been extensive efforts to understand the underlying pathophysiology, and to develop approaches to prevent future occurrences. Currently, the United States Olympic Committee recommends a history, physical exam and screening EKG for their athletes [1]. Clearly, this will fail to identify previously asymptomatic individuals carrying life-threatening structural heart disease that predisposes to sudden cardiac death events, such as that of Flo Hyman. It is also unclear if screening protocols should be tailored to specific sports. For example, are the cardiovascular risks the same for a basketball player as for a gymnast? To address this need, in 2011 we implemented a cardiac screening program for the US Olympic Volleyball national team members that includes a history, physical, and a transthoracic echocardiogram combined with genetic testing on select individuals. In this review I summarize our experience of screening 150 US Olympic National team members and how cardiac imaging combined with genetic testing can help identify at-risk individuals, with the hopes of preventing such tragedies from occurring in the future.

## 2. Materials and Methods

The primary purpose of our screening program is to identify individual athletes that are at risk for sudden cardiac death due to underlying structural heart disease. Specifically, this entails non-invasive imaging of the heart, i.e., transthoracic echocardiography, as we have described previously [2]. Transthoracic echocardiography will identify cardiomyopathies, aortopathies and anomalies of the coronary arteries, among the three most common causes of sudden cardiac death in young athletes.

The Institutional Review Board of the University of California San Diego approved the study as a community service project. The studies were performed on site at the US Olympic Volleyball training facility in Anaheim, California using a portable ultrasound machine by a pediatric board-certified cardiac sonographer. The studies were read by a board-certified pediatric cardiologist through our Intersocietal Commission for the Accreditation of Echo Labs (ICAEL)-certified protocols at Rady Children’s Hospital of San Diego. The history and physical were performed by a board-certified pediatric cardiologist. Although there were some previously reported normal values for aortic measurements for athletes, there had not been any normal values defined specifically for tall athletes until recently. To address this problem, Engel, et al., [3] reported normal values on 526 male NBA players, average height 6’7”, and determined that the upper limit of normal for the sinuses of Valsalva by transthoracic echocardiography was 42mm. More recently, they have reported their echocardiographic findings on 140 female members of the WNBA [4]. Accordingly, we have used 42mm as our criterion for the upper limit of normal for the male athletes and 35mm for females. We also included numerous additional standard measurements to identify other common causes of sudden cardiac death including hypertrophic and dilated cardiomyopathies and anomalous origins of the coronary arteries, as we have described previously [2].

## 3. Results

Since 2011 we have performed cardiac evaluations on 150 athletes. No athletes were found to have echocardiographic evidence for cardiomyopathy or anomalous coronary arteries. We identified five athletes (three males, two females) with mild to moderate dilation of the sinuses of Valsalva, based on the criteria listed above. None of these athletes fulfilled the revised Ghent diagnostic criteria for Marfan syndrome, based on clinical assessment. Athlete #1 is a 23 year-old female with a history of mild scoliosis which resolved, and has no other extra-cardiac findings of Marfan syndrome. She had borderline dilation of the sinuses of Valsalva (3.5cm) and was tested for a Fibrillin-1 mutation, which was negative. She was started on Losartan, 100mg daily for three years, during which time she did not have any progression of her aortic dilation. She elected to discontinue losartan after three years and subsequently retired from the national team. Athlete #2 is a 24 year-old female with mild scoliosis, a positive thumb sign, striae of the skin, pes planus, and a decreased upper to lower body segment ratio. She had mild dilation of her sinuses of Valsalva (3.6cm) with slight progression to 3.7cm over seven years. She had a commercially available 22 gene aortopathy panel, which was negative. She continues to play on the national team. Athlete #3 is a 24 year-old male, with skin striae but no other extra-cardiac findings of Marfan syndrome. He had moderate dilation of the sinuses of Valsalva (4.5cm). Targeted DNA sequencing of the Fibrillin-1 gene revealed a likely disease-causing de novo loss of function mutation [5]. He subsequently retired from playing competitive volleyball. He was started on Losartan, 100mg daily and has had slight progression of his sinuses of Valsalva from 4.5 to 4.7cm over the ensuing seven years. He is contemplating elective surgical repair of his aortic root in the near future. Athlete #4 is a 27 year-old male with a mild pectus excavatum and striae of the skin. He had borderline dilation of the sinuses of Valsalva, measuring 4.2cm. Two years later his sinuses measured 4.3cm, at which time he retired from competitive volleyball. He had the 22 gene aortopathy panel performed, which was negative. Athlete #5 is a 37 year-old male with no extra-cardiac findings of Marfan syndrome. He had mild dilation of the sinuses of Valsalva, measuring 4.3cm initially, and 4.4cm one year later, at which time he retired. He declined genetic testing.

## 4. Discussion

As described above, our screening program has identified five out of 150 (3.3%) athletes with mild to moderate dilation of the sinuses of Valsalva. Genetic testing identified one athlete, the one with the most significant enlargement of the sinuses of Valsalva, with a likely disease-causing gene mutation in Fibrillin-1. He subsequently retired and will likely undergo elective surgical repair in the near future. The three others that underwent genetic testing have mild dilation of their sinuses of Valsalva and have not demonstrated any significant progression of their aortic measurement, although athlete #4’s follow-up was only for two years whereas the other two athletes have had follow up of five and seven years. These three cases suggest the possibility that their mild aortic enlargement is not pathologic. Alternatively, they may have a milder form of a connective tissue disorder for which a specific genetic etiology has not been identified. The other athlete (#5) with mild aortic dilation has not undergone genetic testing and has only had limited follow-up. Similarly, it is unclear whether his dilation is indicative of pathology or a variation of normal.

In summary, genetic testing has proven to be useful in our cohort of athletes with aortic dilation. A positive gene test confirmed our suspicion that one athlete indeed has Marfan syndrome, which has led to potentially lifesaving interventions. The other three athletes that underwent genetic testing had negative results, and their aortic measurements remain only mildly enlarged without any significant progression. Consequently, in these cases negative genetic testing results may provide further reassurance that their mild aortic dilation is not pathologic and will not progress, thereby facilitating clinical decision-making including whether they can continue to participate in competitive volleyball. Regardless, these athletes will require long-term follow-up.

## 5. Future Directions

Our experience combining non-invasive imaging with genetic testing has proven to be useful in identifying at least one player not previously suspected of having Marfan syndrome. An ongoing challenge is how to manage the “phenotype positive/genotype negative” individual. Clearly, serial imaging studies are useful to monitor for progression, which would be indicative of underlying pathology. More robust genetic testing, either an expanded “aortopathy” panel as the number of potentially disease-causing genes continues to increase, or whole genome sequencing may provide further clarity for the identification of at-risk individuals. In the future, it is likely that all newborn infants will undergo whole genome sequencing. This could prove to be useful for identifying at-risk individuals early in life. However, identification of genotype positive/phenotype negative individuals would likely create confusion regarding clinical management. In particular, it confounds whether and when such individuals should be restricted from specific high intensity physical activities, and whether and when medical therapy should be initiated. Genetic testing can also influence the timing of elective surgical repair. Mutations in the TGFβR1 gene, the cause of Loeys-Dietz syndrome, have been associated with a more aggressive progression of aortic dilation and might justify earlier elective repair. In other cases it is less clear when to intervene surgically. For example, when should a male volleyball player with a Fibrillin-1 mutation with an aortic measurement of 4.5cm, i.e. 3mm above the upper limit of normal and not progressing, have surgery? It should also be noted that currently, the NBA prohibits anyone from playing that has the formal diagnosis of Marfan syndrome, regardless of their aortic measurements. However, in some cases players that have undergone elective aortic root replacement that do NOT fulfill the revised Ghent criteria for Marfan syndrome, have been allowed to return to play. Consequently, genetic testing may influence the determination of eligibility to play for elite athletes. Finally, our experience is consistent with previous studies demonstrating aortic enlargement is not uncommon in tall individuals. This raises the question of whether all tall individuals should undergo non-invasive cardiac imaging and if so, at what age and how frequently. At least one such precedent exists for which widespread screening can lead to lifesaving interventions, specifically colonoscopy for the detection of colon cancer [6]. As we accumulate more data for aortopathies in tall individuals, a similar case could be made for the utility of using our combined screening approach for all tall individuals. We are hopeful that our cardiac screening program can be implemented for others besides the small population of elite volleyball players, in order to prevent such tragic and untimely deaths like that of Flo Hyman. Currently, the optimal age for initiation of screening and the frequency that screening should be performed, are unclear.

## Figures and Tables

**Table:** 

#	Sex	Age	Extra-cardiac	Sinuses of Valsalva*	Other cardiac	Genetic testing
11	F	23	Hx of mild scoliosis	3.5,3.6(5)	None	FBN-1 negative
2	F	25	mild scoliosis, positive thumb sign, striae, pes planus, decrease upper to lower segment ratio	3.5,3.7(7)	Normal	Negative panel
3	M	24	striae	4.5,4.7(5)	Normal	FBN-1 mutation^
4	M	27	Mild pectus excavatum, striae	4.2,4.3(2)	Normal	Negative panel
5	M	36	None	4.3,4.4(1)	Normal	None

* Measurements are of the sinuses of Valsalva in centimeters, as described previously (Davis CK, et al., 2015). The first measurement is the initial measurement, followed by the most recent measurement. The number in parentheses refers to the number of years of follow-up since the initial evaluation.

^ 1846G>C; Chr15: 48797336; p.Glu616Gln), which substitutes a highly conserved residue in an epidermal growth factor-like (EGF-like) domain. His parents were tested and did not carry the variant, consistent with a de novo mutation.

## References

[1] Maron B, Thompson P, Ackerman M, Recommendations and Considerations Related to Preparticipation Screening for Cardiovascular Abnormalities in Competitive Athletes: 2007 Update. Scientific Statement American Heart Association 115 (2007): 1643–1655. 10.1161/CIRCULATIONAHA.107.181423 17353433

[2] Davis CK, Dyar DA, Vargas LA, Grossfeld PD. Cardiovascular and Musculoskeletal Assessment of Elite US Volleyball Players. Clin J Sport Med (2015). 10.1097/JSM.0000000000000178 25756701

[3] Engel DJ, Schwartz A, Homma S. Athletic Cardiac Remodeling in US Professional Basketball Players. JAMA Cardiol 1 (2016): 80–87. 27437659 10.1001/jamacardio.2015.0252

[4] Shames S, Bellow NA, Schwartz A, Homma S, Patel N, Echocardiographic Characterization of Female Professional Basketball Players in the US. JAMA Cardiol 5 (2020): 991–998. 32936269 10.1001/jamacardio.2020.0988 PMC7315385

[5] Herrick N, Davis C, Vargas L, Dietz H, Grossfeld P. Utility of Genetic Testing in Elite Volleyball Players With Aortic Root Dilation: Clinical Implications. Medicine and Science in Sports and Exercise (2017). 10.1249/MSS.0000000000001236 PMC5474143 28240702

[6] Sonnenberg A, Cost-effectiveness of colonoscopy in screening for colorectal cancer. Annals of Internal Medicine (2000): 573–584. 11033584 10.7326/0003-4819-133-8-200010170-00007

